# Genotyping of Fanconi Anemia Patients by Whole Exome Sequencing: Advantages and Challenges

**DOI:** 10.1371/journal.pone.0052648

**Published:** 2012-12-20

**Authors:** Kerstin Knies, Beatrice Schuster, Najim Ameziane, Martin Rooimans, Thomas Bettecken, Johan de Winter, Detlev Schindler

**Affiliations:** 1 Department of Human Genetics, University of Wuerzburg, Wuerzburg, Germany; 2 Department of Clinical Genetics, Vrije Universiteit (VU) Medical Center, Amsterdam, The Netherlands; 3 Center for Applied Genotyping Munic, Max-Planck-Institut für Psychatrie, Munich, Germany; Charité Universitätsmedizin Berlin, NeuroCure Clinical Research Center, Germany

## Abstract

Fanconi anemia (FA) is a rare genomic instability syndrome. Disease-causing are biallelic mutations in any one of at least 15 genes encoding members of the FA/BRCA pathway of DNA-interstrand crosslink repair. Patients are diagnosed based upon phenotypical manifestationsand the diagnosis of FA is confirmed by the hypersensitivity of cells to DNA interstrand crosslinking agents. Customary molecular diagnostics has become increasingly cumbersome, time-consuming and expensive the more FA genes have been identified. We performed Whole Exome Sequencing (WES) in four FA patients in order to investigate the potential of this method for FA genotyping. In search of an optimal WES methodology we explored different enrichment and sequencing techniques. In each case we were able to identify the pathogenic mutations so that WES provided both, complementation group assignment and mutation detection in a single approach. The mutations included homozygous and heterozygous single base pair substitutions and a two-base-pair duplication in *FANCJ*, -*D1*, or -*D2*. Different WES strategies had no critical influence on the individual outcome. However, database errors and in particular pseudogenes impose obstacles that may prevent correct data perception and interpretation, and thus cause pitfalls. With these difficulties in mind, our results show that WES is a valuable tool for the molecular diagnosis of FA and a sufficiently safe technique, capable of engaging increasingly in competition with classical genetic approaches.

## Introduction

Fanconi anemia (FA) is an autosomal or X-chromosomal recessive disorder characterized by variable yet typical developmental malformations, bone marrow failure and predisposition to leukemia and solid tumors. As much as 15 genes define corresponding complementation groups designated as FA-A, -B, -C, -D1, -D2, -E, -F, -G, -I, -J, -L, -M, -N, -O and -P. Biallelic or in the case of FA-B hemizygous mutations in any one of the underlying genes lead to FA, while monoallelic mutations in *FANCD1* (*BRCA2*), *FANCJ* (*BRIP1*), *FANCN* (*PALB2*) or *FANCO* (*RAD51C*) increase the risk of carriers for developing breast and ovarian cancer [1]. FA patients commonly suffer from physical abnormalities like short stature, abnormal skin pigmentation, radial ray defects and malformations of the ears, eyes and inner organs. More than 80% of FA patients develop progressive bone marrow failure which makes pancytopenia a highly suggestive clinical feature [2]–[4]. In addition, FA patients show not only greatly elevated frequencies of myelodysplastic syndrome and acute myeloid leukemia in childhood, but there is also markedly increased prevalence of non-hematologic malignancies. They experience an up to 700-fold higher risk of squamous cell carcinomas especially of the head and neck or anogenital region [3], [5]. Other solid tumors are less frequent among FA patients but a variety of them are still extraordinarily common compared to the general population [5]. The reason for the overall increased cancer risk may be due to the DNA repair defect that characterizes the cellular phenotype [6]. FA cells show elevated rates of chromosomal breakage and typical radial rearrangement figures. These features occur spontaneously but are exaggerated following exposure of cultured cells to DNA crosslinking agents such as diepoxybutane (DEB) or mitomycin C (MMC) [7]–[9]. Since this hypersensitivity is typical for FA cells, chromosomal breakage analysis is used as a diagnostic tool. Alternatively, cell cycle studies or cellular survival assays using flow cytometric methods are utilized for diagnosis because FA cells are hindered to pass the G2 checkpoint control, accumulate in G2 phase of the cell cycle and show increased death rates after DNA damage induction [10], [11]. On the molecular level diagnosis is more complicated. Even though about 60% of FA patients carry mutations in *FANCA* [3], 14 other FA and several associated genes remain that may contain disease-causing defects. While there is so far no cure for FA, knowledge of the individual complementation group and the specific mutations of patients may be important for differential, prenatal or preimplantation diagnosis, prognosis or upcoming gene therapy trials. Biallelic mutations of *FANCD1*, for example, are associated with early-onset acute myelogenous leukemia and blastomas [12], [13]. Subtyping of FA patients can be performed by cell fusion experiments, retroviral complementation analysis or in some cases by Western blotting, but the specific mutations have to be analyzed by Sanger sequencing. Because of the high number of FA and FA-associated genes and because some of these genes have more than 40 exons, DNA sequencing by Sanger technique is becoming increasingly tedious, time-consuming and costly.

Recently, an efficient and reliable technique, Next Generation Sequencing (NGS), emerged to improve and accelerate conventional methods of molecular diagnostics. In the present study we demonstrate the versatility of Whole Exome Sequencing (WES) in four independent projects. The four patients involved had previously been confirmed to be afflicted with FA by non-molecular procedures but were not assigned to any complementation group and thus lacking accountable mutations. Using WES we genotyped each patient by the identification of their disease-causing mutations in one of three different FA genes. Thus we consider WES an efficient tool to compete with traditional approaches for the molecular diagnosis of FA.

## Materials and Methods

### Study design

The study scope, patient information and consent form were approved by the Ethical Review Committee of the Medical Faculty of the University of Wuerzburg.

### Cell cycle analysis

For confirmation or exclusion of FA we used flow cytometric cell cycle analysis as described earlier [11], [14].

### DNA sample preparation

Genomic DNA was isolated from patient-derived fibroblasts (patients 1, 3 and 4) using the *GeneJet™* Genomic DNA Purification Kit (Fermentas, patients 1 and 3) or the QIAamp DNA isolation kit (Qiagen, patient 4) following the manufacturer's instructions. For isolation of gDNA from peripheral blood of patient 2–1, his siblings 2–2 and 2–3 and their parents we used a salting-out technique.

### Whole Exome Sequencing

Enrichment and sequencing of the exomes of projects 1 to 3 were commissioned to different service providers on an exclusively commercial basis. Sample 1 was enriched by means of the *NimbleGen SeqCap EZ Human Exome Library v2.0* and sequenced *on* an *Illumina HiSeq2000*. For sample 2 the *Agilent SureSelect Human All Exon 38 Mb Kit* (hg18) was used together with the *SOLiD* sequencing technology by *Applied* Biosystems. In project 3 WES was performed with *SOLiD4* technology after enrichment using the *Agilent SureSelect Human All Exon 50 Mb Kit* (hg19). Raw data from *Illumina* sequencing were provided in *fq* format. *SOLiD* raw data were provided in *csfasta* format along with *qual* files containing corresponding quality information. For project 4 we used the *SureSelect Human All Exon Kit* (Agilent) targeting approximately 38 Mb, following the manufacturer's instructions. The enriched sample was sequenced on one lane of the *Illumina GAIIx* instrument using a paired-end sequencing protocol, which is available upon request.

### Data analysis

Analysis of the WES data of projects 1 to 3 was performed using the alignment and analysis software *NextGENe™* v2.18 by *Softgenetics*. The raw data were filtered for low quality reads before alignment. Based on the enrichment kits being used, reads that passed the quality filter were aligned to the whole human genome hg18 in project 2 and hg19 in projects 1 and 3. The average exome coverage was determined using a complete list of human exons generated by the *UCSC Table Browser*. The same procedure was performed for FA gene coverage. The following analytical steps were performed only with reads that matched exonic regions including exon-intron-boundaries. SNP and insertion/deletion (indels) analysis was done by different filtering steps depending on whether consanguinity was suspected or not. In patient 2–1 of consanguineous descent only homo-/hemizygous variants were taken into account. In patients 1 and 3 with non-consanguineous background genes with at least two heterozygous changes in the DNA sequence were considered to be most likely disease-causing, even though homozygous variants were not completely withdrawn.

For sample 4 we used a data analysis pipeline for the evaluation of single nucleotide variants and small indels, which was comprised of tools freely available on the web. The paired-end reads were mapped by the Burrows-Wheeler Aligner (BWA) [15] to the reference genome built according to NCBI hg19. Subsequently, SNPs and small indels were called using Samtools [16] and Varscan [17]. The resulting list of variants was annotated with Annovar [18] that summons and utilizes information from external databases to assess implications and consequences of a given sequence alteration, such as an ensuing amino acid change, location within a canonical splice site, and information from dbSNP along with the SNP frequency if available. Finally, a manual filtering step was carried out to prioritize relevant mutations.

### Filtering strategy

Holding for all samples, the variant detection frequency was set at a minimum of 20% of the reads covering any aberration. A minimum coverage by 10 reads was set as threshold for any variant to be considered a real mutation. In each case all variants listed in the most recent version of the NCBI (National Center for Biotechnology Information) dbSNP database were excluded as well as silent mutations. Low frequency frameshift and truncating mutations in any FA gene were considered pathogenic. Unreported non-synonymous amino acid variants were analyzed *in silico* by Align-GVGD (data not shown), MutationTaster (http://www.mutationtaster.org), Polyphen-2 (http://genetics.bwh.harvard.edu/pph2) and SIFT (http://sift.jcvi.org) to assess any potentially damaging effect. Variants passing these filtering steps were considered to be most likely disease-causing and forwarded to validation process by Sanger sequencing and other techniques.

### Sanger sequencing

Potential mutations were verified by Sanger sequencing generally using an *Applied Biosystems 3130xl* instrument. Primer sequences and PCR conditions are available upon request.

### Immunoblotting

FANCD2 expression analysis was performed with whole protein extracts isolated from patient-derived fibroblasts. Cell lines were treated with hydroxyurea or MMC before analysis. We used primary antibodies including mouse monoclonal anti-FANCD2 (sc20022, Santa Cruz Biotechnology), mouse monoclonal anti-RAD50 (GTX70228, GeneTex), rabbit polyclonal anti-RAD51 (ab63801, Abcam), rabbit polyclonal anti-FANCJ (NB 100-416A, Novus) and mouse monoclonal anti-Vinculin (sc-25336, Santa Cruz Biotechnology). Secondary antibodies included Alexa Fluor 594 goat anti-rabbit IgG (H+L) (A11012, Invitrogen), Goat pAb to rabbit IgG (HRP) (ab97200, Abcam), Donkey pAb to mouse IgG (HRP) (ab98665, Abcam).

### Autozygosity mapping

Autozygosity mapping was performed with SNP data generated with the Illumina SNP array HumanHap300v2. Genotypes were analyzed using AutoSNPa software [19].

## Results

### Confirmation of the FA diagnosis

**Figure 1:**
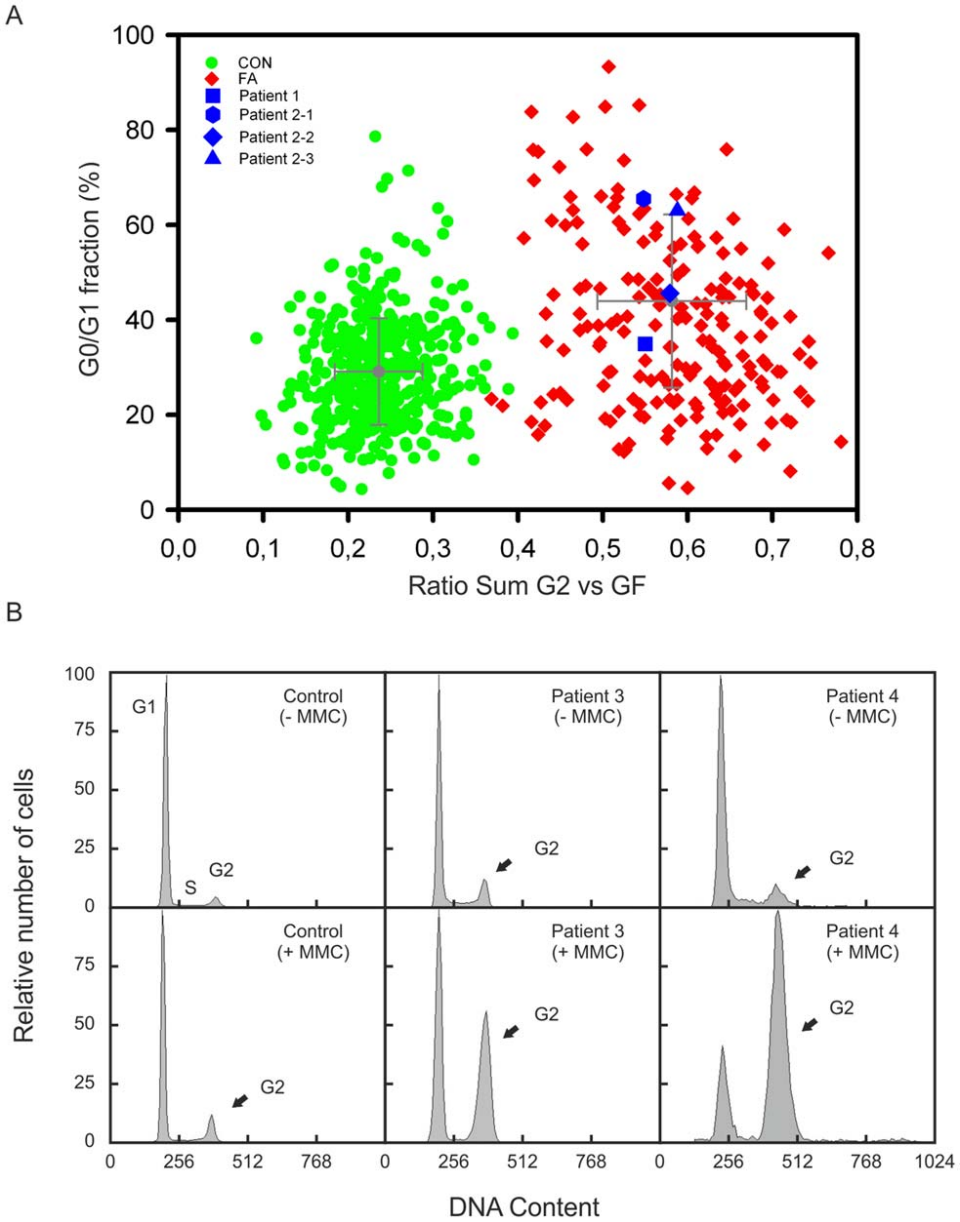
Cell cycle analysis. (A) Graphic presentation of the results of flow cytometric cell cycle analysis. Peripheral blood lymphocytes were exposed to MMC. The ratio “sum of all G2 phases vs. growth fraction” was calculated from individual cultures and plotted against the G0/G1 phase compartment. Cells from patient 1 and the siblings 2–1, 2–2 and 2–3 show high ∑G2/GF ratios (blue squares) similar to those from other persons with FA (red diamonds), but were distinct of normal controls (green dots). (B) Flow histograms of fibroblast cultures from patients 3 and 4 show increased G2 arrest after exposure to MMC, in contrast to a control cell line (arrows).

In each project the clinical diagnosis was confirmed by flow-cytometric cell cycle analysis. In patients 1 and 2 FA was evident from studies of peripheral blood lymphocyte cultures. After 72 h incubation with 10 ng/ml MMC the ratio “sum of all G2 phases vs. growth fraction” was above 0.4 which is characteristic of FA patients (Fig. 1A) [20]. Patients 3 and 4 showed distinct cell accumulations in the G2 phase (>20%) in fibroblast cultures exposed to 12 ng/ml MMC for 48 h, likewise consistent with other FA patients (Fig. 1B).

### Mutation detection by WES and validating experiments

Statistical data of each WES project are summarized in Table 1 and Table S2. The exome was covered on average between 22x and 77x. The FA and FA-associated genes with homozygous or at least two potentially heterozygous mutations were first assessed. Assignment of the mutations to different alleles and screening for their pathogenicity revealed the following results.

**Table 1:** Summary of statistical data from four independent WES projects.

	Project 1	Project 2	Project 3	Project 4
Total read number	33,661,920	121,791,357	152,961,886	27,371,419
Average read lenght	86bp	45bp	40bp	72bp
Reads passing QC	32,251,042	82,558,019	117,526,556	27,371,419
Reads on target (whole exome)	29,837,615 (93%)	67,361,646 (82%)	83,597,787 (71%)	20,707,708 (76%)
Reads on target (FA genes)	17,518	93,806	76,257	31,925
Average exome coverage	22x	77x	71x	36x
Average coverage of FA genes	21x	56x	53x	31x
Total number of variants	20,065	13,466	14,978	18,885
Known SNPs/MNPs	15,213	9,846	13,563	15,469
UV in cs	4,652	3,567	1,386	3,281
UV at ess	200	46	29	136
Homozygous UV (cs+ess)	107	201	44	30
Heterozygous UV (cs+ess)	4,745	3,419	1,371	3,387
Silent UV (cs)	1,012 het	73 hom	286 het	884
Missense UV (cs)	2,736 het	102 hom	920 het	2,289
Nonsense UV (cs)	113 het	1 hom	44 het	23
Unknown InDels (cs+ess)	766 het	24 hom	101 het	77
Multiply heterozygous mutated genes	683	-	207	411
Homozygous mutated genes	-	102	-	-

QC, quality control; UV, unknown variants; cs, coding sequence; ess, essential splice sites; SNP, single nucleotide polymorphism; MNP, multiple nucleotide polymorphism.

The initial number of genes with homozygous or double heterozygous mutations was counted without filtering for low mutation scores or benign sequence changes.

#### Project 1

**Figure 2:**
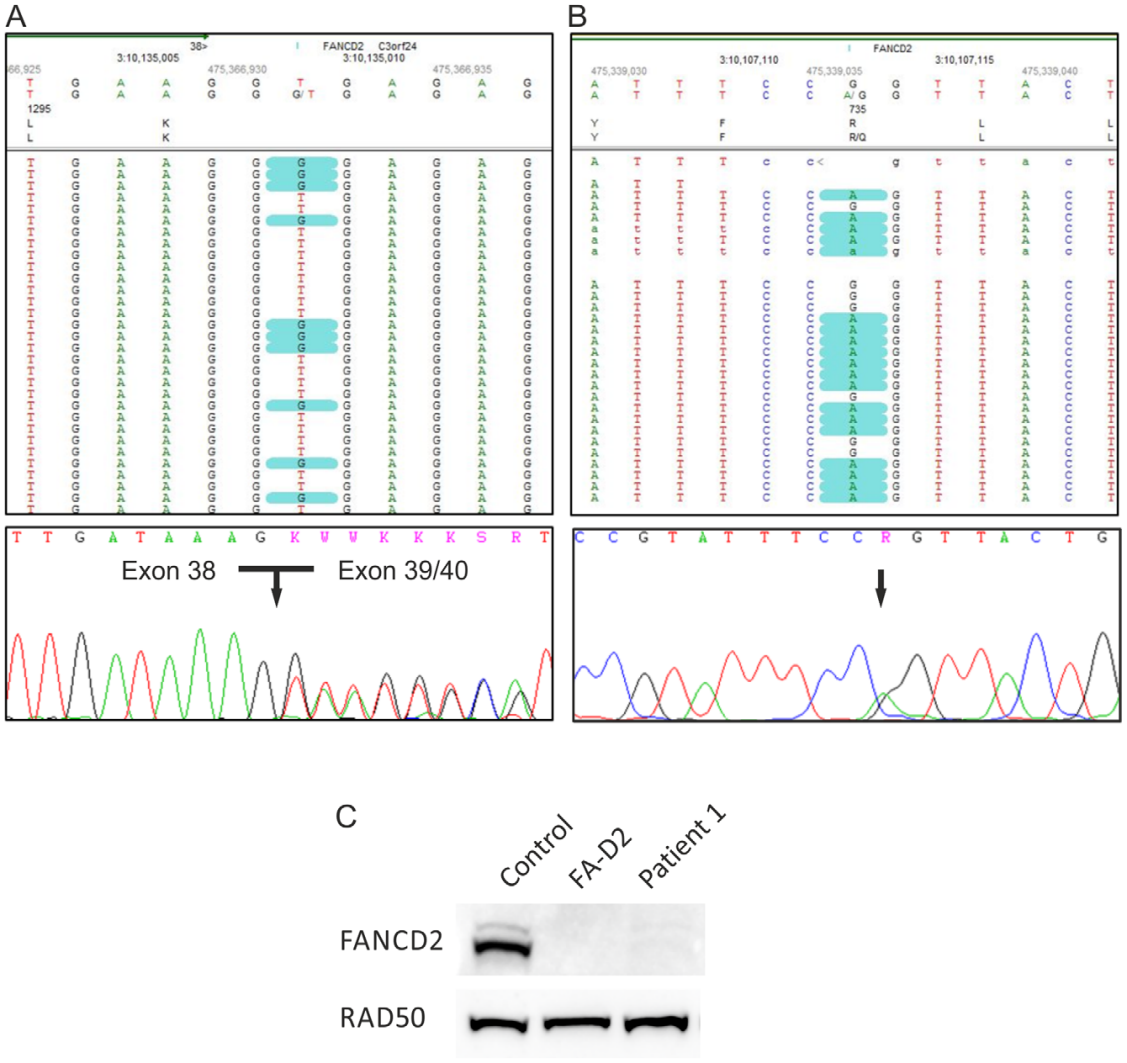
Genotyping of patient 1. (A) The heterozygous *FANCD2* splice site mutation c.3888+2T>G in patient 1. The upper panel demonstrates mutation calling in NGS data format. The lower panel shows an electropherogram of Sanger cDNA sequencing depicting heterozygous skipping of exon 39. (B) The heterozygous *FANCD2* missense mutation c.2204G>A. The upper panel demonstrates the substitution in NGS data format, while the lower panel shows the confirmation by Sanger sequencing of gDNA. (C) An immunoblot shows very faint FANCD2-S and -L bands after exposure of fibroblasts from patients 1 to MMC (lane 3). This was similar to other FA-D2 patients (example on lane 2) but contrasted markedly to normal controls (example on lane 1). RAD50 was used as loading control.

We employed 3 µg gDNA isolated from cultured fibroblasts of patient 1 to enrich the whole exome. WES revealed two heterozygous mutations with a score ≥10 (probability 1∶100 for being false positive) exclusively in *FANCD2*. They included the single-base substitution c.2314G>T, resulting in a premature stop codon at amino acid position p.772, and the canonical splice site change c.3888+2T>G in exon 38. Sanger sequencing confirmed the splice site alteration in gDNA and showed an *in frame* skipping of exon 39 at the cDNA level (Fig. 2A). Because of the pseudogene *FANCD2-P2*, containing an incomplete copy of the active gene region [21], validation of the nonsense mutation by Sanger sequencing was performed on long range PCR product (exon 22 to exon 26). Re-sequencing of this super-amplicon did not confirm the substitution c.2314G>T (Fig. S1A). Thus we concluded that this variant had occurred in the pseudogene and therefore could not be causative of FA in that patient. By decreasing the filter settings we additionally detected the missense substitution c.2204G>A in exon 24 resulting in the amino acid change p.R735Q. Even though this base is alsopresent in the pseudogene sequence, its assessment by gene-specific super-amplification rendered it an authentic *FANCD2* mutation (Fig. 2B). We confirmed the maternal segregation of p.R735Q, whereas the splice site change was not present in maternal gDNA and may have occurred *de novo* or was, more likely, inherited from the father, of whom no material was available. Finally, decreased abundance of FANCD2 protein in the patient's cells confirmed our DNA sequencing results (Fig. 2C).

#### Project 2

**Figure 3:**
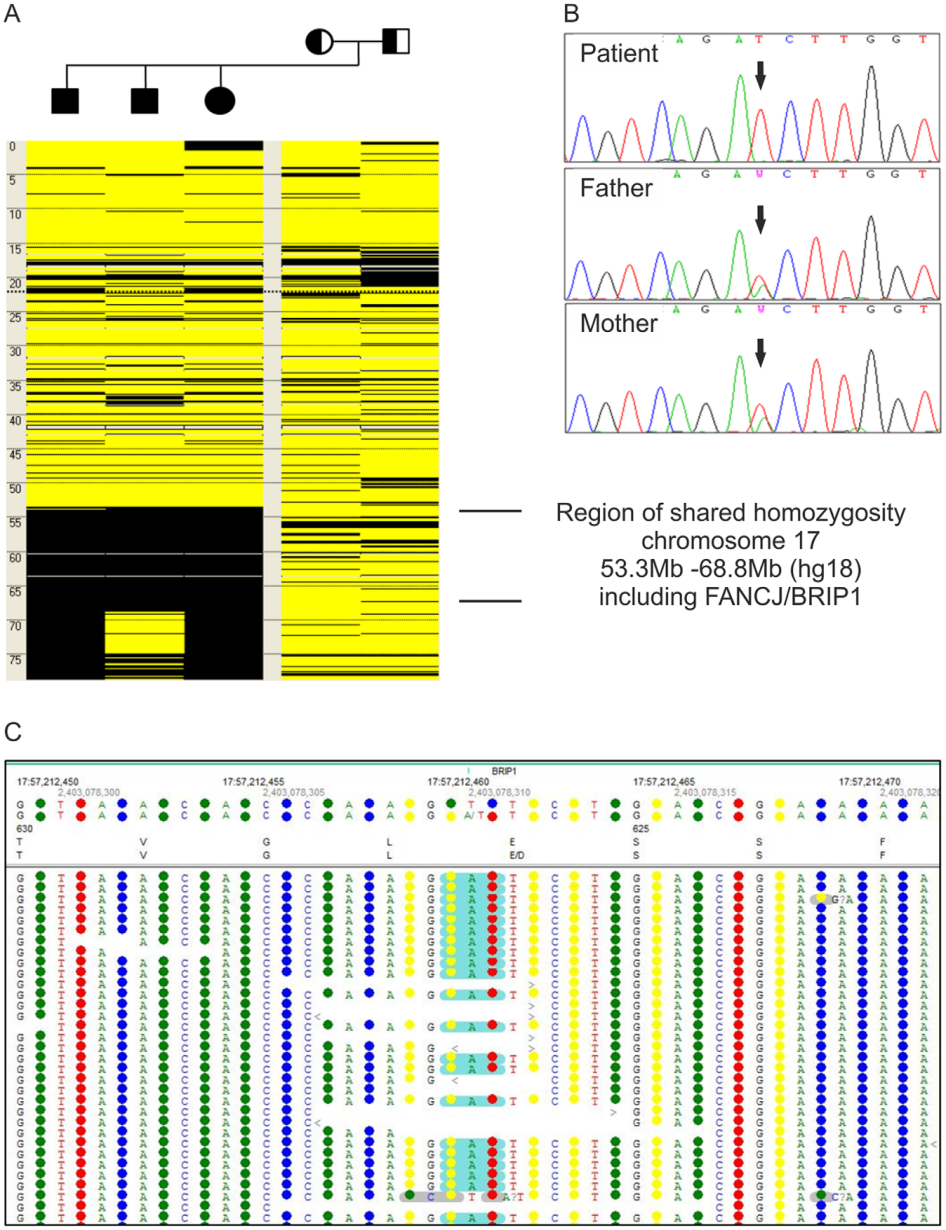
Genotyping of patient 2. (A) Homozygous mutation call c.1878A>T in *FANCJ* detected in NGS data of patient 2–1. (B) Autozygosity mapping with SNP data of the family of project 2. The figure schematically presents chromosome 17 (positions in Mb) of each family member. Heterozygous SNP calls are displayed in yellow, homozygous calls in black. The three affected siblings share a homozygous region between 53.3 Mb and 68.8 Mb. (C) Confirmation of homozygosity of the mutation in patient 2–1 and heterozygosity in his parents by Sanger sequencing electropherograms, consistent with Mendelian segregation.

Parallel to WES we performed a genome-wide SNP study in patient 2–1, his two affected siblings (2–2, 2–3) and their parents. Autozygosity mapping using these data revealed a large homozygous region on chromosome 17 (Fig. 3A). The whole exome was enriched from 3 µg gDNA, isolated from peripheral blood of patient 2–1. Analysis of the color-spaced *SOLiD4* sequencing data revealed the homozygous single base substitution c.1878A>T in exon 13 of *FANCJ* (Fig. 3B) compatible with the outcome of the disease gene mapping. This point mutation results in the amino acid change p.E626D (Fig. 3B) that is predicted to be pathogenic (Table S1). Sanger sequencing confirmed the homozygous mutation of the patient (Fig. 3C) and his siblings (data not shown). These results and the heterozygous detection of the mutation in both parents (Fig. 3C) were consistent with Mendelian segregation. Additionally we could detect reduced FANCJ protein levels by Western blot analysis of whole protein extracts from patient derived-cell lines (data not shown).

#### Project 3

**Figure 4:**
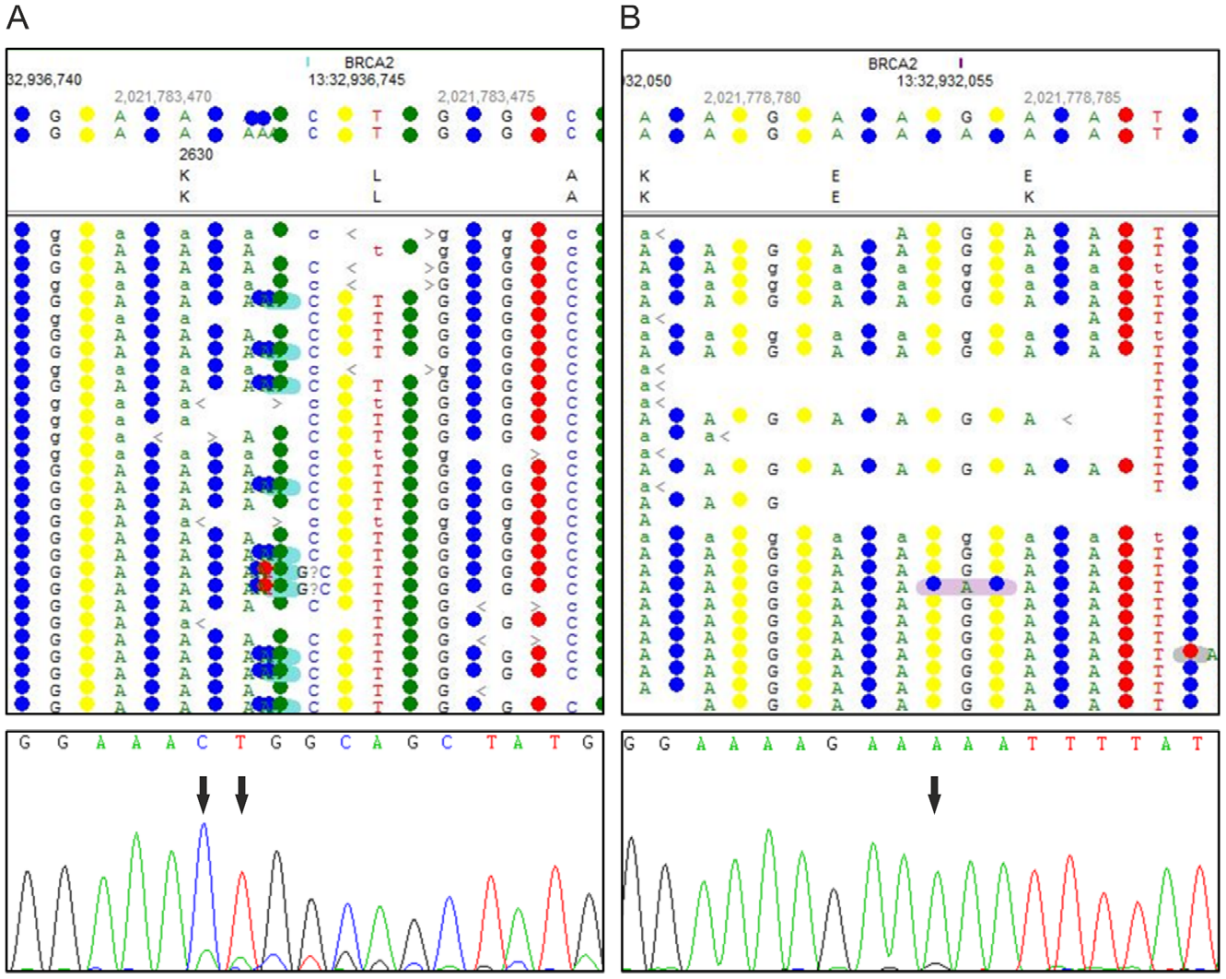
Genotyping of patient 3. (A) NGS data in the upper panel show the insertion c.7890_7891insAA in *FANCD1* detected in patient 3. The electropherogram in the lower panel demonstrates corresponding validation by Sanger sequencing. (B) The upper panel misleadingly displays the single-bp substitution c.7795G>A in the NGS data of patient 3 as a SNP, highlighted in pink. Confirmation by Sanger sequencing is shown below.

3 µg gDNA of patient 3 were isolated from fibroblasts. Initial analysis of the WES data failed to show FA or FA-associated genes with biallelic mutations. Re-examination of all unknown variants and listed SNPs resulted in the identification of a 2-bp insertion c.7890_7891dupAA in exon 17 of *BRCA2*/*FANCD1* (Fig. 4A) with the effect p.L2631Nfs16X. Three additional variants were found in the same gene, of which only c.7795G>A was predicted to be pathogenic (Fig. 4B, Table S1). The SNP rs80359682 listed at this position is a deletion of three bases (c.7795_7797delGAA) in exon 16, which is of unknown pathogenicity, whereas our detected single-bp substitution results in the probably damaging missense mutation p.E2599K. We confirmed both mutations of patient 3 by Sanger sequencing, even though the allele carrying the insertion was detectable only at a very low level (Fig. 4A). In accordance with those results we detected proficient FANCD2 monoubiquitination and impaired RAD51 foci formation in the patient derived fibroblastic cell line (data not shown).

#### Project 4

**Figure 5:**
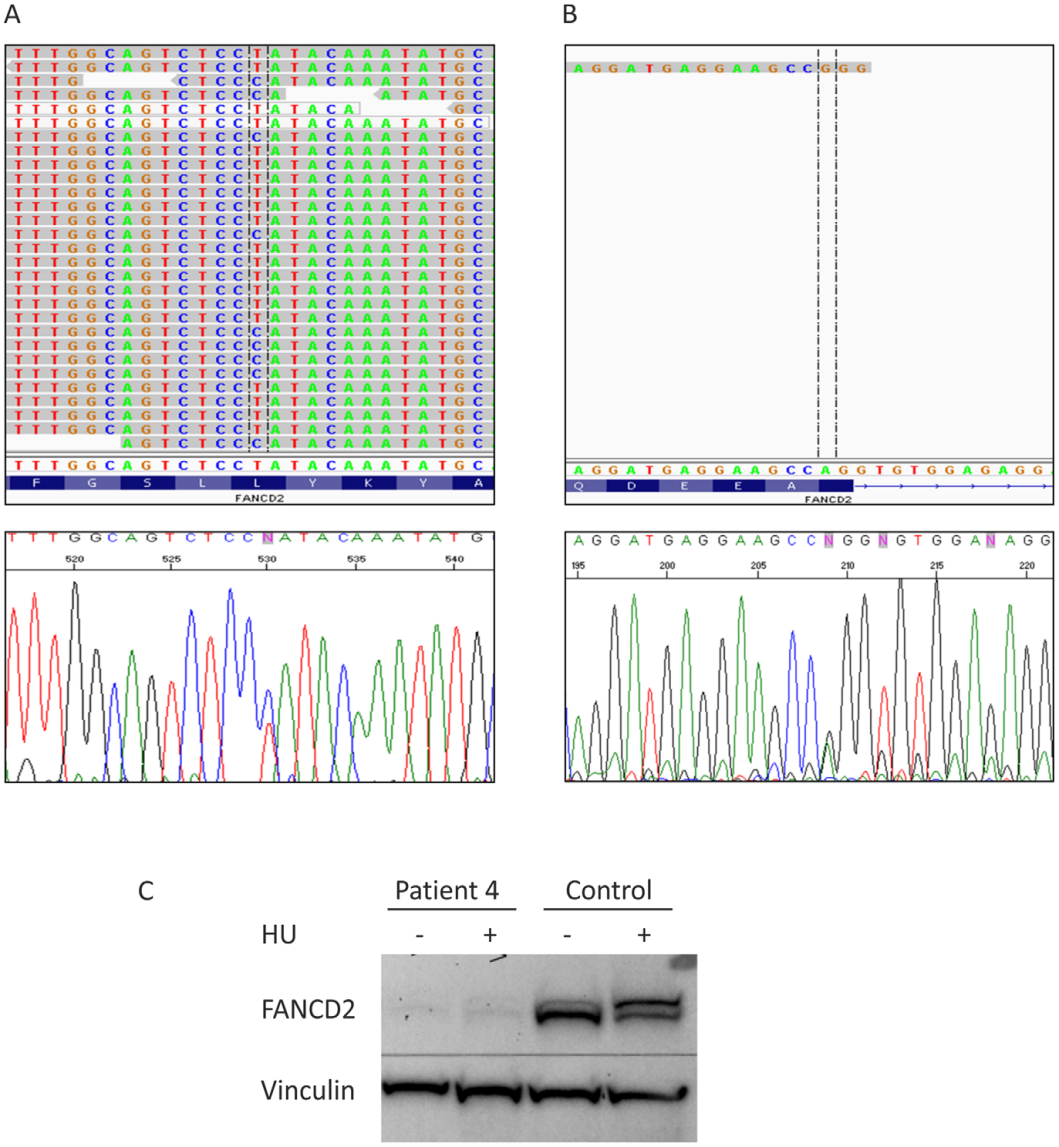
Genotyping of patient 4. (A) Displayed is the *FANCD2* mutation c.1370T>C in patient 4 in NGS data as well as validated by Sanger sequencing. (B) The upper panel shows NGS data with low coverage of *FANCD2* exon 5 containing the substitution c.376A>G. The electropherogram in the lower panel depicts validation by Sanger sequencing. (C) Hydroxyurea (HU) treated (+) and untreated (−) fibroblasts of patient 4 show very low levels of both the S and L species of residual FANCD2 protein. Vinculin was used as loading control.

In contrast to projects 1 to 3, where data have been analyzed by means of the alignment and analysis software *NextGENe™* v2.18 by *Softgenetics*, we used for project 4 an in-house variation detection pipeline to score sequence variants [21]. We focused on rare variants within the coding and splice site regions of all known FA genes. Only one already reported heterozygous base substitution was detected in *FANCD2* exon 16, c.1370T>C (p.L457P), which had previously been recognized to be pathogenic [22]. Initially we failed to detect a second mutation in *FANCD2*. After visual inspection of the mapped reads in the IGV browser, *FANCD2* exon 5 was shown to be covered by a single read. Therefore, the data appeared unreliable for mutation detection. Subsequent Sanger sequencing of that exon demonstrated a c.376A>G base substitution resulting in another missense mutation p.S126G, that had previously been shown to be pathogenic and affects splicing [23] (Fig. 5A). Western blotting revealed distinct deficiency of the FANCD2 protein (Fig. 5B).

## Discussion

The present study proposes the application of WES for the molecular diagnosis of FA. Major concerns with WES are ethical issues, less its performance. Potentially, WES data could be used to analyze any gene, or even all genes, for any purpose. In our projects the patients had given informed consent for FA diagnostics. We addressed and resolved the discrepancy to WES in the way that we used the whole body of data only for quality control, statistical analyses, and to apply general filtering settings. For mutation screening we solely regarded FA and FA-associated genes.

We performed four independent sequencing projects with disparate exome enrichment and sequencing technologies. Although the performances differed slightly, we were able to identify the disease causing mutations in all index patients. Except for the mutations in patient 4, all of the detected pathogenic variants had not yet been listed in the *Fanconi Anemia Mutation Database* (http://www.rockefeller.edu/fanconi/) such that we consider the identification of missense, nonsense and splice site mutations as well as a 2-bp insertion in *FANCD1, FANCD2* and *FANCJ* by WES a challenging task and major accomplishment. The successful outcome led us to conclude that WES generally is a reliable tool for the molecular diagnosis of FA. It also proved to be efficient in time and adequate in expense. Including sample quality control, target enrichment, sequencing and basic bioinformatics each of our projects was brought to completion within two to three months. Alignment and mutation calling afforded another few days, followed by validation processes. The cost of each of the four projects was highly variable and ranged from 800$ to 4500$, but decreased in tendency over time. Sanger sequencing of all FA genes would have exceeded the projects' current term and cost several times. Sanger sequencing of the 43 FANCA exons only would be comparable to the price of exome sequencing but would fail to detect the mutation in more than 40% of cases. Enrichment of the FA gene regions followed by NGS would be an effective alternative, because it could increase the locus-specific coverage and decrease cost and sequencing time. Although this approach has been published recently [21], so far this application is not commercially available for the FA genes. In some instances WES holds some advantages compared to a target enrichment approach, because there are still FA patients who cannot be assigned to any of the reported complementation groups. In agreement with the patient WES data can be used for further screenings not only including FA-associated genes such as FAAP100, but also of other candidate genes. An additional agreement between patient and the diagnostic lab addressing this issue is required.

In all presented cases we found point mutations or small insertions to be disease-causing. Therefore we can safely assume that those patients do not carry additional large insertions or deletions, which would have been difficult to identify by WES. In the case of large and complex variations this technical drawback can, however, be avoided if WES is combined with MLPA or microarray techniques. Additionally a recent report by Ameziane et al. (2012) detected large deletions after NGS of enriched FA gene regions by evaluating the Log2 ratio of the local read depth divided by a read depth reference [21]. None of the FA patients in our four projects had mosaicism in the hematopoietic system as shown by diagnostic procedures preceding WES. If there was indication for such a situation it would have been appropriate to use fibroblast DNA for WES. Deep intronic mutations, which could affect splicing, might be the only kind of sequence changes that are not detectable by WES. However those mutations are rare in FA and would anyway be difficult to identify by classical approaches. Our study also raised technical issues and revealed methodical difficulties that should be addressed. For each project we analyzed the exome coverage and in particular the coverage of the FA genes (Table 1). Even though the average exome coverage in the *SureSelect* enrichment projects clearly exceeded the coverage in the *NimbleGen* project, we found coverage of the FA gene regions in the latter to be more consistent and complete (Table S2). A similar observation was reported by Clark et al. (2011) for the whole exome in general. Most of the entirely unsequenced exons in our study had a high GC or high AT content leading to the conclusion that excess GC content is still a limiting factor for efficient hybridization and amplification during target enrichment [24]. In project 4 initially only one pathogenic mutation was detected, while the other mutation was missed because of insufficient coverage. In this case the GC and AT content of *FANCD2* exon 5, where the second mutation later was identified, is balanced with 44% and 56%, but probably the high AT content of the adjacent intron regions that were included in enrichment may explain the low coverage. Nevertheless, the identification of the first mutation led to close examination of that gene and subsequent identification of the second mutation. On the other hand it is a rare but recurrent experience that a single heterozygous mutation in one FA gene may accompany compound heterozygous, disease-causing mutations in another FA gene.

We observed a lower rate of sequencing errors in *SOLiD* data. The two-base-encoding technology leads to lower rates of false positive or false negative base calls and facilitates the discrimination of sequencing errors from authentic mutations [25], [26]. Incomplete or even contradictory gene databases can complicate the validation process and can cause confusion by wrong and incomplete or misleading mutation calling. In project 3 we experienced a problem even with SNP databases. A truly pathogenic mutation was designated as a SNP because there was a known polymorphism that included the mutated base pair. To avoid such pitfalls as far as possible and because mutation screening becomes easier the more polymorphisms are excluded, we recommend using always the latest version of the dbSNP database in combination with minor allele frequencies and information from other sources such as the 1000 Genomes project.

Another issue to consider during *in silico* pathogenicity assessment is the choice of mutation prediction software. For the mutations described in this study we compared the performance of three different mutation prediction tools (*SIFT, PolyPhen-2* and *MutationTaster*). While *SIFT* and *Polyphen-2* often failed to ascertain the pathogenic effect of the mutations, *MutationTaster* generally was able to provide a reliable prognosis for all genes and every type of mutations.

Finally, in this and other NGS studies we noticed that the existence of pseudogene sequences can complicate the detection of genuine mutations residing in functional genes and thus may result in false positives. In project 1 re-sequencing showed that the c.2314G>T mutation call in *FANCD2* was due to incorrect mapping of the variant containing reads, which should have mapped to the pseudogene, *FANCD2-P2*. The missense mutation c.2204G>A likewise represented *FANCD2-P2* pseudogene sequence. In this case it proved to be a true *FANCD2* mutation at the same time. In that same exon we identified two more base substitutions representing pseudogene sequence but the corresponding reads were misleadingly mapped to *FANCD2*. Only gene-specific re-sequencing resolved the correct sequence (data not shown). We recognized this problem not only in FA genes. For example, another project had revealed a hemizygous deletion including the *CDC27* locus, but WES unexpectedly showed heterozygous base variants of that gene. On closer inspection we found three related pseudogenes, containing the complete cDNA sequence of *CDC27* from exon 3 to 14. This led us to re-check the putative gene variants by Sanger sequencing. All of them turned out to be false positives attributable to pseudogene sequences (data not shown). We suggest that this problem may be due to the short read length produced by *SOLiD* and *Illumina* NGS and ambiguous mapping during alignment with the genome. Pseudogenes are characterized by high sequence similarity with their corresponding functional genes and therefore ambivalent mapping in the analysis of NGS data cannot always be avoided. In terms of FA genes, special attention needs to be paid to *FANCD2* for which only *FANCD2-P1* LOC100421239 is listed in the NCBI database but not the other reported pseudogene, *FANCD2-P2* [22]. For *FANCL* and the FA-associated gene *MHF1* at least partial copies have been disclosed.

Notwithstanding the challenges with WES data analysis, we would recommend it as a valuable tool for FA genotyping. In our opinion, WES, if carefully applied, is able to compete with classical molecular approaches in diagnostics and research not only for FA but generally for other disorders with locus heterogeneity.

## Supporting Information

Figure S1 **Validation by Sanger technique.** (A) Sanger sequencing of cDNA revealed a false positive result of c.2314 G>T in *FANCD2* being a mutation of patient 1 due to interference with the pseudogene *FANCD2-P2*. (B) Confirmation of Mendelian segregation of c.2204 G>A and c.3888+2 T>G. The missense mutation is inherited from the mother. The healthy sister is a heterozygous mutation carrier. The canonical splice site change must have occurred *de novo* or been inherited from the father whose DNA was not available. It was not detectable in other family members.
(DOCX)

Table S1 **Predicted effect of the mutations detected in** ***FANCD1*** **,** ***FANCD2*** **and** ***FANCJ.***
(DOCX)

Table S2 **Coverage of FA genes.**
(DOCX)
