# Supplementary figures and images for: Genotyping of Fanconi Anemia Patients by Whole Exome Sequencing: Advantages and Challenges
Source: PLoS One. 2012 Dec 20;7(12):e52648. doi: 10.1371/journal.pone.0052648 (PMC3527584; doi:10.1371/journal.pone.0052648)

**Supporting information**

**Figure S1**

**
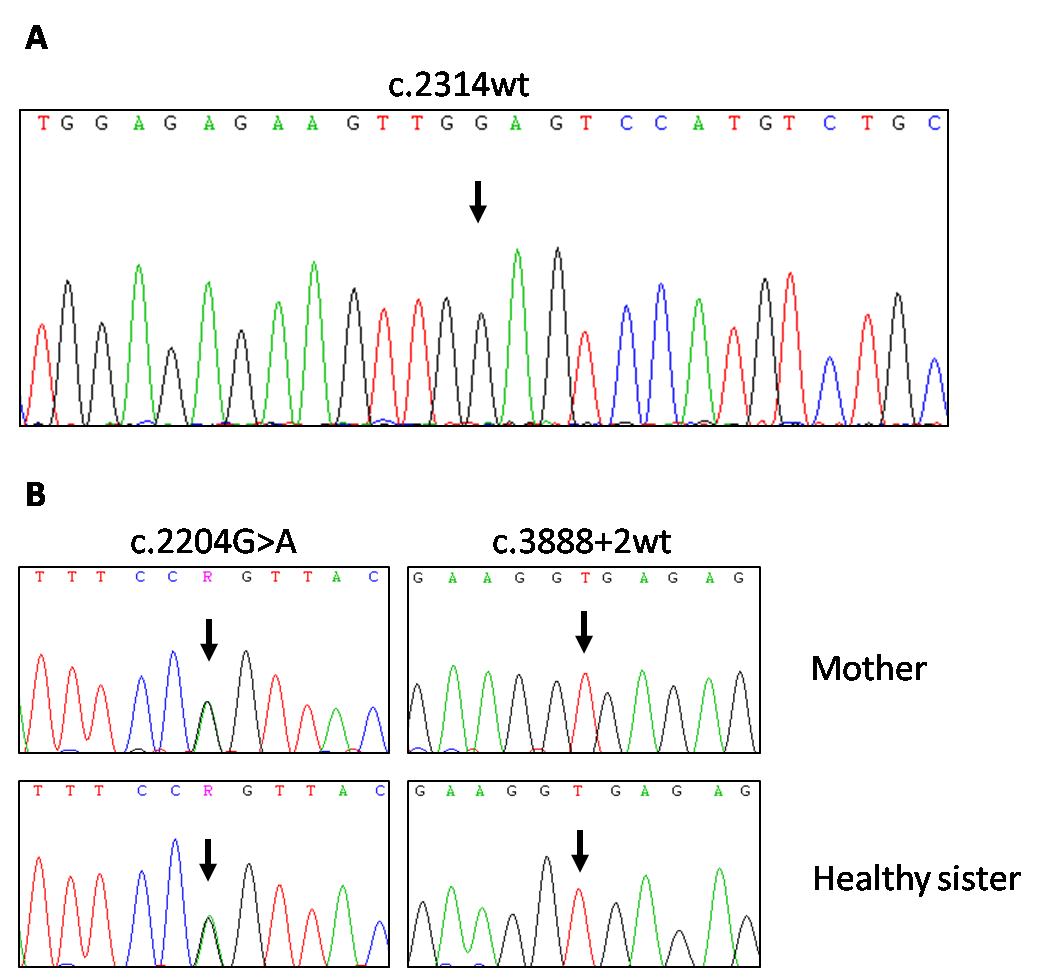
**

Supplement: Figure S1 — Validation by Sanger technique. (A) Sanger sequencing of cDNA revealed a false positive result of c.2314 G>T in FANCD2 being a mutation of patient 1 due to interference with the pseudogene FANCD2-P2. (B) Confirmation of Mendelian segregation of c.2204 G>A and c.3888+2 T>G. The missense mutation is inherited from the mother. The healthy sister is a heterozygous mutation carrier. The canonical splice site change must have occurred de novo or been inherited from the father whose DNA was not available. It was not detectable in other family members. (DOCX) [file pone.0052648.s001.docx]
