# Supplementary material for: Genotyping of Fanconi Anemia Patients by Whole Exome Sequencing: Advantages and Challenges
Source: PLoS One. 2012 Dec 20;7(12):e52648. doi: 10.1371/journal.pone.0052648 (PMC3527584; doi:10.1371/journal.pone.0052648)
Supplement: Table S1 — Predicted effect of the mutations detected in FANCD1 , FANCD2 and FANCJ. (DOCX) [file pone.0052648.s002.docx]

**Supporting information**

**Table S1.** Predicted effect of the mutations detected in *FANCD1*, *FANCD2* and *FANCJ*

|  | **Mutation Taster** | **Poly-Phen 2** | **SIFT** |
| --- | --- | --- | --- |
| **FANCD2**  **c.2204G>A** | 55% disease causing | 100% probably damaging | damaging, score 0.01 |
| **FANCD2**  **c.3888+2T>G** | 72% Polymorphism,  Splice Site Change | - | - |
| **FANCD2**  **c.1370T>C** | 86% Polymorphism | 100% probably damaging | damaging, score 0 |
| **FANCD2**  **c.376A>G** | 74% Polymorphism | 100% probably damaging | damaging, score 0.02 |
| **FANCJ**  **c.1878A>T** | 71% disease causing | 100% probably damaging | - |
| **FANCD1**  **c.7890insAA** | 100% disease causing,  NMD, FS | - | - |
| **FANCD1**  **c.7795G>A** | 83% disease causing,  rs80359682 | - | damaging, score 0.01  rs 80359682 |

The percentages denominate probability of being pathogenic.
