# Supplementary material for: Genotyping of Fanconi Anemia Patients by Whole Exome Sequencing: Advantages and Challenges
Source: PLoS One. 2012 Dec 20;7(12):e52648. doi: 10.1371/journal.pone.0052648 (PMC3527584; doi:10.1371/journal.pone.0052648)
Supplement: Table S2 — Coverage of FA genes. (DOCX) [file pone.0052648.s003.docx]

**Supporting information**

**Table S2.** Coverage of FA genes.

|  | **Project 1** | **Project 2** | **Project 3** | **Project 4** |
| --- | --- | --- | --- | --- |
| **FANCA** | 1 (79%) | 1 (79%) | 1 (79%) | 1 (79%), 22 (63%) |
| **FANCB** |  |  |  |  |
| **FANCC** |  |  |  |  |
| **FANCD1** |  | 12 (31%) |  |  |
| **FANCD2** |  |  |  | 5 (44%) |
| **FANCE** | 1 (76%) | 1 (76%) | 1 (76%) | 1 (76%) |
| **FANCF** |  |  |  |  |
| **FANCG** |  |  |  |  |
| **FANCI** |  | 23 (36%) |  | 23 (36%) |
| **FANCJ** |  |  |  |  |
| **FANCL** |  |  |  |  |
| **FANCM** |  |  |  |  |
| **FANCN** |  |  |  |  |
| **FANCO** |  |  |  |  |
| **FANCP** |  |  |  | 8 (72%) |
|  |  |  |  |  |

Listed are the completely unsequenced exons of 15 FA genes in the four independent WES projects. Corresponding GC contents are given in percent.
